# Assessment of the Polychlorinated Biphenyl (PCB) Occurrence in Copper Sulfates and the Influential Role of PCB Levels on Grapes

**DOI:** 10.1371/journal.pone.0144896

**Published:** 2015-12-14

**Authors:** Xiaomin Li, Xiaoou Su

**Affiliations:** Institute of Quality Standard and Testing Technology for Agro-Products, The Chinese Academy of Agricultural Sciences (CAAS), Beijing, 100081, China; University of Siena, ITALY

## Abstract

Copper sulfates (CuSO_4_) are widely used as the primary component of fungicides in the grape industry. The agricultural-grade CuSO_4_ that we collected from Chinese nationwide markets were found to be contaminated by polychlorinated dibenzo-*p*-dioxins and dibenzofurans and high levels of polychlorinated biphenyls (Σ_19_PCBs: 0.32~9.51 ng/g). In the following research, we studied the impact of CuSO_4_ application on PCB levels in grape products through a field experiment, and conducted a national survey to speculate the role that CuSO_4_ played on the occurrence of PCB in grapes. In the field experiment, an obvious increase of PCBs in grape leaves (from 174 to 250 pg/g fw) was observed after Bordeaux mixture (the main component of which is CuSO_4_) application. As to the main PCB congener in CuSO_4_, the most toxic CB 126 (toxic equivalency factor = 0.1) also increased in grape peels (from 1.66 to 2.93 pg/g fw) after pesticide spray. Both the correlation study and the principal component analysis indicated that environmental factors were dominant PCB contributors to grapes, and grapes from e-waste dismantling area containing the highest PCBs also proved the notion. It is worth noting that this report describes the first research examining PCBs in CuSO_4_ and its influence on agricultural products to date.

## Introduction

Both polychlorinated biphenyls (PCBs) and polychlorinated dibenzo-*p*-dioxins and dibenzofurans (PCDD/Fs) are known as notorious persistent organic pollutants (POPs) and were listed into the Stockholm Convention [1]. The concise structure of these chemicals, two benzene rings modified with chlorine and linked directly or with an ether bond, makes them highly stable and likely to be generated during organochlorine-related chemical industry [2–3]. The toxicity of PCBs, especially dioxin-like PCBs (dl-PCBs), is similar to PCDD/Fs. The PCBs are collectively referred to as dioxin-like compounds, and toxic equivalent (TEQ) has been adopted to assess the health risk aroused by PCDD/Fs and dl-PCBs [4]. It has been determined that the levels of PCBs are generally orders of magnitude higher in the environment than PCDD/Fs due to their high-volume production [5–7]. Once used as effective dielectric fluids in capacitors and transformers, nearly 10,000 tons of PCBs were produced during 1965~1974, of which 90% were trichlorobiphenyls [8–9]. PCDD/Fs are never intentionally produced.

Copper sulfate (CuSO_4_) is widely used in agriculture. It is not only applied as an effective fungicide to fruits and plants, but is also used as a feed additive in the animal feeding industry and as a type of metal fertilizer. The Bordeaux mixture (BM: the main content of this fungicide is a proportional mixture of CuSO_4_ and quicklime with water), for instance, is widely used as a type of fungicide to work against downy mildew that commonly occurred on grapes (*Vitis vinifera L*.). Recently, Wang et al. recognized CuSO_4_ as a potential PCDD/Fs-contaminated industrial product. More than half of CuSO_4_ samples contained PCDD/Fs exceeding EU limitation[10]. As aforesaid, BM is used many times during fruit grown and mature periods. If the CuSO_4_ was contaminated by these pollutants, they might discharge to the environment and find its way into plants or directly stick on the surface of grapes and then enter the human body through ingestion. Thus, the application of BM might pose a potential health risk to humans through consuming grapes.

Several studies have noticed the dioxin-like compounds in vegetables and fruits. Papadopoulos et al. collected apples, grapes, oranges, peaches, and pears in Greek markets and found that the total TEQs of PCDD/Fs and non-ortho-PCBs ranged from 0.38 to 0.55 pg WHO-TEQ g^-1^ [11]. In a later review, the data showed an average 0.05 pg WHO-TEQ g^-1^ dw of PCDD/Fs in Spain, and fruits contributed 3.1% to the total intake [12]. Normally, dietary exposure research attributes higher ingestions of POPs to animal products, such as fish, meat and milk, especially in the western countries [13–14]. Fruits were considered less contaminated by POPs; however, fruits and vegetables contributed very large portions of the daily intake in China, which took up more than 17% and 30% of the total food consumption, respectively [15]. Therefore, far more attention needs to be paid to PCBs ingestion through grape consuming in these countries.

PCDD/F existence might positively be related to PCB existence in many industrial processes [16–17]. PCDD/Fs were once identified in CuSO_4_; however, the PCBs in CuSO_4_ have not been published so far. In the present study, we first collected a batch of agricultural-grade and analytical-grade copper sulfate on the Chinese market and determined both the levels of PCDD/Fs and PCBs. Second, we selected a vineyard as our field experiment site and collected soil, air, leave, and fruit samples during the growing cycle for the grape to investigate the influence of BM application and the environmental factors on dioxin-like compounds in grapes. Considering that CuSO_4_ was widely applied around the country, a national survey of PCB concentrations in grapes was further conducted to evaluate whether the PCB contamination in CuSO_4_ was a universal problem or not. This study might shed light on whether it is safe to eat grapes considering their most extensively and abundantly used pesticide was a dioxin-like compound-contaminated industrial product.

## Materials and Methods

### 2.1. Sample Collection and Preparation

Ten agricultural grade CuSO_4_ (C1~C10) samples were collected from the market or vineyard in different provinces. To make a comparison, four analytical-grade CuSO_4_ (C11~C14) samples were simultaneously collected.

**Fig 1:**
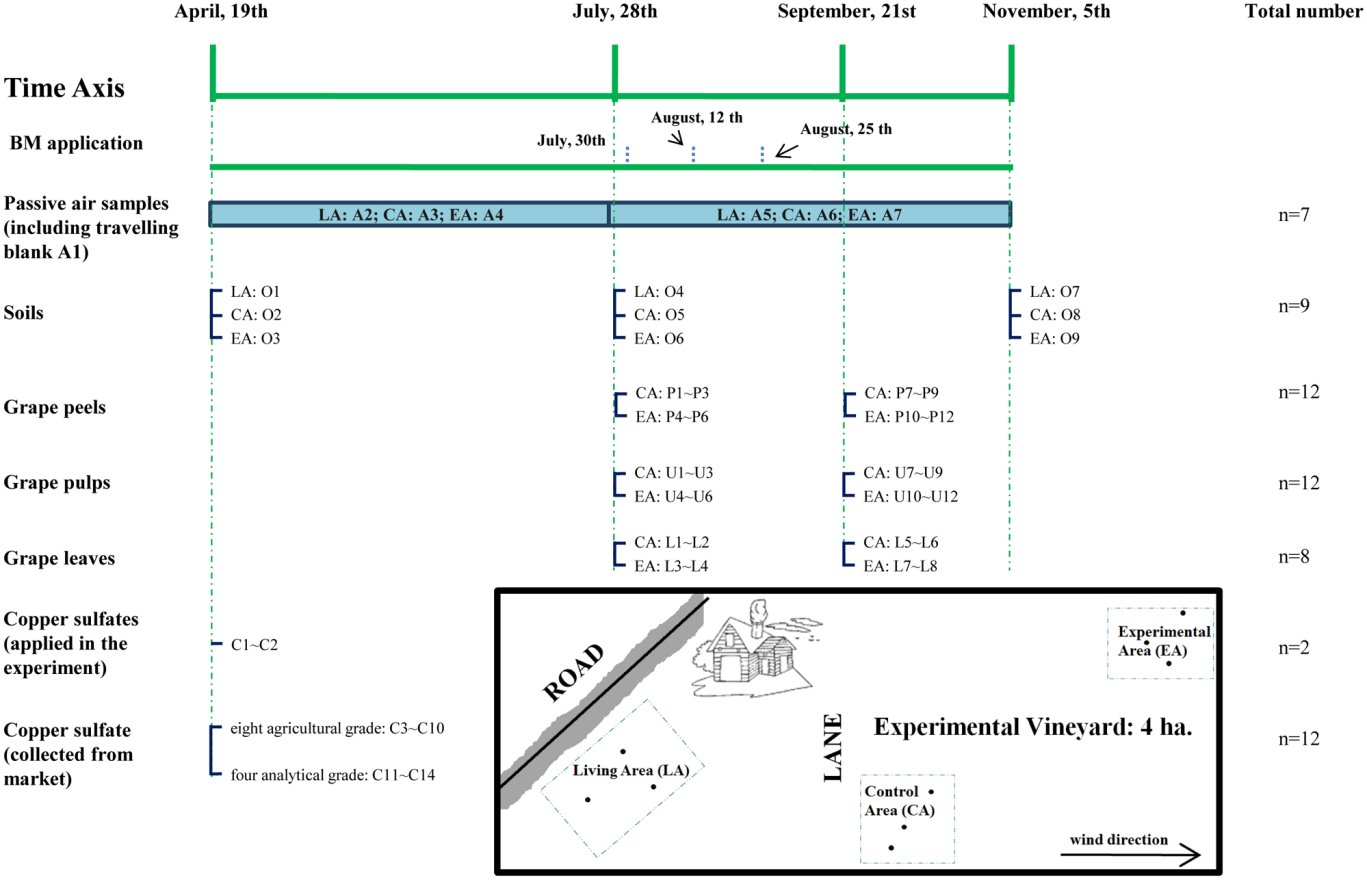
Descriptions of sampling project and corresponding sample IDs in the field experiment.

The sample descriptions, sampling dates and sample numbers of the field experiment are shown in Fig 1. Details of the sampling schedule and information about each sample were given in Table A in S1 File. The vineyard was located northwest of Beijing, and this field has only been used as a vineyard for two years. The sampling zones were divided into the living area (LA), the control area (CA), and the experimental area (EA) (Fig 1). Both CA and EA were in the vineyard, the difference being that no BM was sprayed in the CA this year. The experimental period lasted from April 19th to November 5th, 2013. The prevailing wind direction was from the south during our sampling period. The CA was set at the south of the EA to avoid the possible influence of BM application. Passive air samplers (concentrated by a polyurethane foam (PUF) disk) were employed in the LA, CA, and EA. The BM was applied three times this year, on July 30th, August 12th, August 25th. Air samples were collected 99 days before and 97 days after the first BM application day. The sampling rate of the PUF was set to 3.5 m^3^/day, which followed a global network using the same sampler [18]. Travelling blanks were prepared, sealed in zip-lock bags and simultaneously analyzed with field air samples. Soil samples were collected at the same time with air samples. A five-point sampling method was adopted.

Leaves and grapes were collected before BM application and 9 days after the final application. A withdrawal period was allowed to better understand the real situation. All the field samples were immediately covered by aluminum foil and sealed in zip-lock bags to transport to the lab. Moreover, pooled CuSO_4_ that were used in this vineyard were also collected and marked as C1 and C2 for further analysis.

To evaluate the target pollutants’ contents in market grapes, we collected 27 grape samples from 21 cities within 11 provinces in China. Most of them were from the major grape production areas (Table B in S1 File), and four grapes were intentionally collected from the contaminated areas.

Grapes were separated into peels and pulps manually. Next, the grape peels, grape pulps, soils, and leaves were lyophilized and homogenized. Water contents were recorded, and all of the data were reported in fresh weight. There was not a significant difference between the moisture contents of the grape peels and pulps, with geometric means of 82.8% and 85.2%, respectively. The average moisture of the leaves was 74.9%. The samples were stored at -18°C until analysis.

### 2.2. Reagents and Materials

^13^C-labeled surrogate standards for PCDD/Fs (EPA 1613-LCS) and PCBs (EPA 68A-LCS) and ^13^C-labeled injection standards for PCDD/Fs (EPA 1613-IS) and PCBs (EPA 68A-IS) were all purchased from Wellington Laboratories (Guelph, Canada). Pesticide-grade dichloromethane and n-hexane were purchased from J.T. Baker (Phillipsburg, USA); nonane was purchased from Sigma-Aldrich (St. Louis, USA). Silica gel (0.063–0.100 mm) was purchased from Merck (Darmstadt, Germany) and baked at 550°C for 12 h before use. Anhydrous sodium sulfate (baked at 660°C for 6 h), concentrated sulfuric acid and sodium hydroxide were of reagent grade (purity>99.8%) and were purchased from Beijing Chemistry Company (Beijing, China). Acid silica gel was made by evenly mixing 70 g of activated silica gel impregnated with 30 g of concentrated sulfuric acid, and basic silica gel was made by mixing 100 g of activated silica gel and 30 mL of sodium hydroxide solution (1 mol L^-1^). Activated carbon was purchased from Supelco (Bellefonte, USA).

PUF disks (diameter: 14 cm; thickness: 1.35 cm) were pre-cleaned by accelerated solvent extraction (ASE350, Dionex, USA) using acetone and a mixture of dichloromethane:hexane (v/v, 1:1) in sequence. Then, they were wrapped with aluminum foil and preserved in sealed plastic bags until use. The other information of reagents and materials were provided in S1 File.

### 2.3. Analytical Method and Quantification

For CuSO_4_, 10 g were extracted using soxhlet extraction with acetone:dichloromethane (v:v, 1:1) for 24 h. All the soxhlet extractions were pre-cleaned with clean solvent for 6 h before using. Both ^13^C-labeled 1613-LCS and 68A-LCS were added to the samples. Multilayer silica columns and activated carbon columns were used to clean up the samples. ^13^C-labeled 1613-IS and 68A-IS were added before instrumental analysis.

Two grams of grape peels or pulps, 1.5 g of leave, or 10 g of soil were dispersed with 10 g of anhydrous sodium sulfate and then extracted by ASE. Before the extraction, ^13^C-labeled 68A-LCS was added. Then, dichloromethane:hexane (v:v, 1:1) was used to extract the samples at 120°C and 1500 psi for 3 cycles. The heating time was 7 min, and the static time was 8 min. For the PUF disks, the extraction temperature was 100°C. The sample cleanup procedure has been described in detail in a previous work [19]; we made minor modifications here. Briefly, the extracts were first pre-cleaned with acid silica gel and then filtered through an anhydrous sodium sulfate column. After concentration, the extract was further cleaned on a multilayer silica column (from bottom to top, packed with 1 g of active silica gel, 4 g of basic silica gel, 1 g of active silica gel, 8 g of acid active silica gel and 2 cm of anhydrous sodium sulfate) and a carbon column (1.5 g of activated carbon mixture and 2 cm of anhydrous sodium sulfate). Then, the extracts were concentrated to approximately 20 μL. ^13^C-labeled 68A-IS was added before instrumental analysis.

In this study, 17 PCDD/F congeners and 19 PCB (including 12 co-planar, 6 indicator and CB 209) congeners were analyzed for CuSO_4_, and 19 PCBs were analyzed for other samples by high-resolution gas chromatography (Agilent 6890, Agilent Tech., USA) coupled with high-resolution mass spectrometry (HRGC/HRMS, Micromass Autospec Ultima, Waters, UK). The GC column was a DB-5MS fused silica capillary column (J&W, Scientific, 60 m, 0.25-μm film thickness, 0.25 mm i.d.). The temperature program for PCDD/Fs began at 150°C, increased to 230°C after 3 min (20°C /min) and held 18 min, then increased to 235°C (5°C /min) and held 10 min, finally increased to 330°C (4°C /min) and maintained for 3 min. The temperature program for PCBs began at 120°C, increased to 150°C after 1 min (30°C /min), then reached 300°C (2.5°C /min) and held 1 min. The resolution of HRMS was more than 10,000. The electron emission energy was 35 eV, and the source temperature was 270°C.

### 2.4. Quality Assurance and Quality Control (QA/QC)

The isotope dilution method was applied to monitor qualification and quantification efficiency. The surrogate standards showed that the average recoveries of the PCBs were in the range of 58.4~115%, and for the PCDD/Fs, the recoveries were between 40.4~66.6%. All these results satisfied the demands of USEPA methods 1613B and 1668A. The travelling blanks and laboratory blanks were inserted into each analytical batch. Anhydrous sodium sulfate was lyophilized with each batch, also acting as a blank. The concentrations of our target compounds in the blanks were under the limit of detection (LOD). The LOD for each congener is defined as a signal-to-noise ratio (S/N) of 3. The LOD of PCBs and PCDD/Fs was in the range of 0.025~0.693 pg/g and 0.033~0.127 pg/g, respectively.

### 2.5. Statistical Analysis

The toxic equivalent (TEQ) values were calculated based on the newest published WHO toxic equivalency factor [20]. Statistical analyses and mathematical calculations were processed with SPSS 19.0 (IBM, USA). Pearson’s correlation analysis was used to analyze the correlations among environmental factors, grape products and copper sulfates. Principal component analysis (PCA) was performed to examine the characteristics of the samples collected after BM spray and the national grape samples. Statistical significance was set to *p*<0.01 unless otherwise specified. For those that were below the LOD, the concentrations were set at zero.

### 2.6 Ethics Statement

No specific permissions were required for the described study, which complied with all relevant regulations.

## Results and Discussion

### 3.1. Concentrations of PCDD/Fs and PCBs in Copper Sulfate

**Fig 2:**
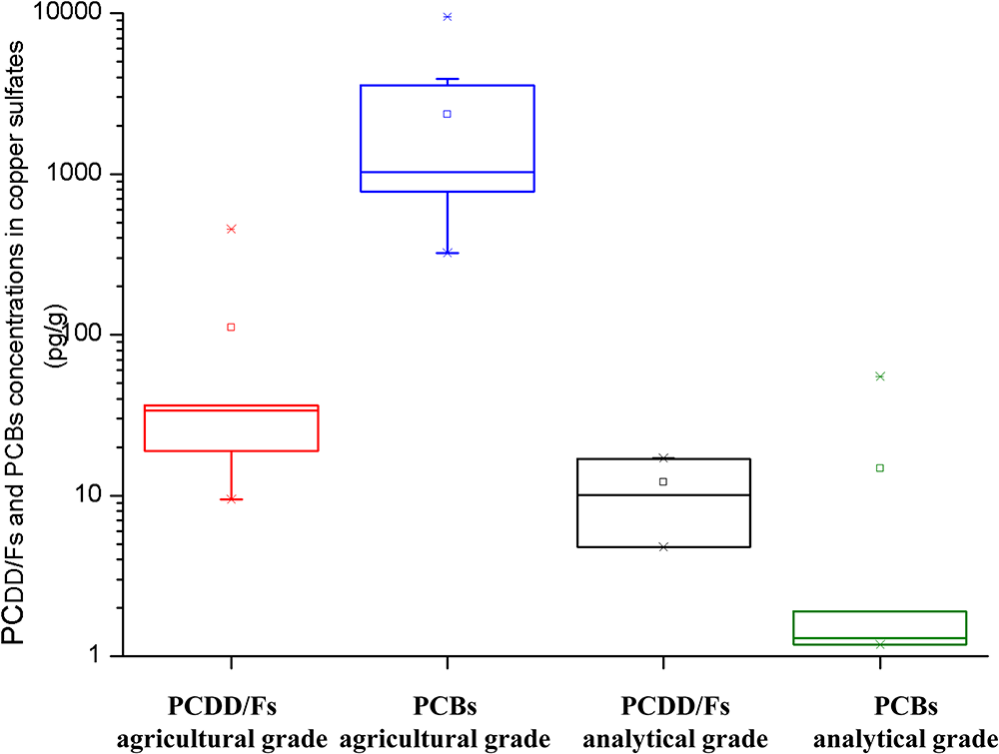
Concentrations of Σ_17_PCDD/Fs and Σ_19_PCBs in agricultural-grade (n = 10) and analytical-grade (n = 4) CuSO_4_. The box upper and under lines represent the 25th and 75th percentiles, respectively, and the three horizontal bars represent the 5th, 50th, and 95th percentiles. * represent the 1% and 99%, □ represent mean values.

The levels and congener patterns of PCDD/Fs and PCBs were totally different between analytical-grade and agricultural-grade CuSO_4_ (Fig 2). The concentrations of Σ_17_PCDD/Fs and Σ_19_PCBs in the 10 agricultural-grade CuSO_4_ (C1~C10) were in the range of 9.47~454 pg/g and 0.32~9.51 ng/g, respectively. The TEQs of C1~C10 were between 6.66 and 106 pg WHO-TEQ g^-1^, with an average of 38.6 pg WHO-TEQ g^-1^. The average level of PCBs in CuSO_4_ was 20 times higher than that of PCDD/Fs, and PCDD/Fs only contributed 1.2~4.0% to the total TEQs in the market CuSO_4_. Wang et al. reported that levels of PCDD/Fs in CuSO_4_ were in the range of 8.58~41.2 pg/g, and TEQ values were 0.35~3.92 pg WHO-TEQ g^-1^, and those compounds came from polluted hydrochloric acid [10]. Our results not only showed higher PCDD/Fs concentrations in CuSO_4_ than Wang et al.’s work but also found much higher PCB contamination in CuSO_4_, as well. Interestingly, the indicator PCBs were not the main contributors to the total PCB mass concentrations in the agricultural-grade CuSO_4_. The co-planar congeners, such as CB 77, 169 and 126, contributed an average of 33.2, 13.1, and 12.6% to the Σ_19_PCBs. CB 126 has been identified as a major contributing factor for the TEQ, accounting for 78.2% in C1~C10. The levels of Σ_17_PCDD/Fs and Σ_19_PCBs in analytical-grade CuSO_4_ (C11~C14) were much lower than those in agricultural-grade CuSO_4_, ranging from 4.75 to 17.0 and 1.30 to 54.8 pg/g, respectively. The TEQs of C11~C14 were between 0.06~0.19 pg WHO-TEQ g^-1^. Furthermore, the homologue distributions of PCBs were quite different between the agricultural-grade CuSO_4_ and analytical-grade CuSO_4_ (Fig A in S1 File). PCDD/Fs were the main TEQ contributors in analytical-grade CuSO_4_, accounting for 96.4~98.8%.

The European Commission has limited the sum of PCDD/Fs and dl-PCBs in feed-grade CuSO_4_ to be less than 1.0 pg WHO-TEQ g^-1^ [21]. There is no regulation specifying the limitation of PCDD/F and PCB concentrations in agricultural-grade CuSO_4_ as of yet; however, if we used EU regulation as a reference, all ten agricultural-grade CuSO_4_ we collected exceeded the limitation. Furthermore, a very strong correlation was observed among the PCB congeners in agricultural copper sulfates (including the field-collected samples (C1~C2) and market samples (C3~C10)). The correlation coefficients of C1~C10 were in the range of 0.777 to 0.975 (*p*<0.005) (Table C in S1 File). The results implied that CuSO_4_ was a wide and common source of PCBs. The susceptibility and universality of CuSO_4_ to dioxin-like compounds should arouse public concern. The PCB-contaminated CuSO_4_ were widely used in agriculture, which might cause universal PCB contamination. Furthermore, a field experiment was conducted to study the influence of CuSO_4_ application on PCB contamination in the grapes.

### 3.2 Field Experiment

C1 and C2 which were used in this field experiment were also the routinely used fungicide in the experimental vineyard. The average concentration of Σ_19_PCBs in C1 and C2 was 860 pg/g, and the TEQ was 16.4 pg WHO-TEQ g^-1^. It is a medium level compared to the market agriculture-grade CuSO_4_. The PCDD/Fs levels in most of the analyzed grape samples were under the LOD, and considering the little contribution of PCDD/Fs in agricultural-grade CuSO_4_ (in C1 and C2, accounted for 6.6% of the total TEQ values), only PCBs were discussed in the following contents.

**Fig 3:**
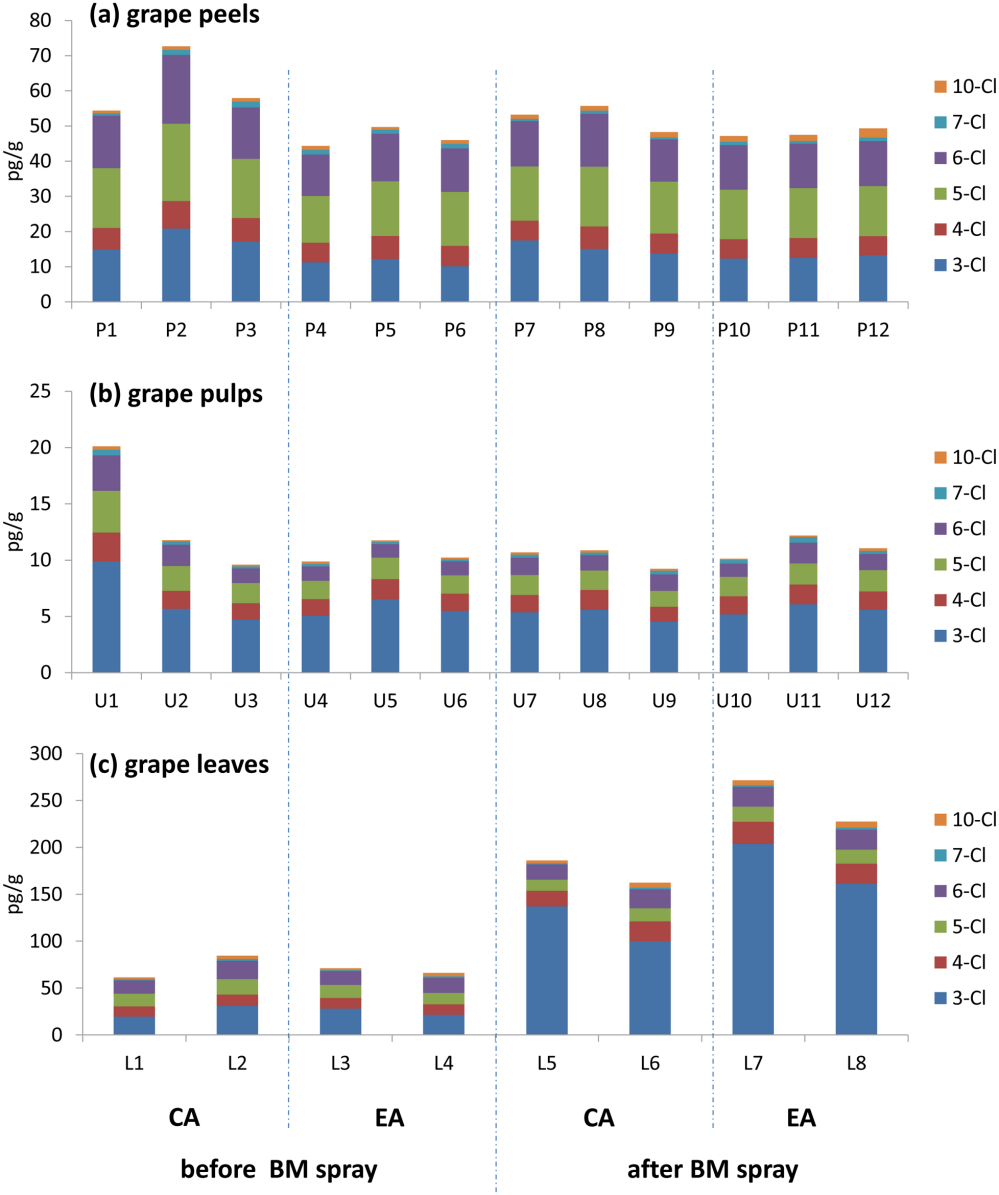
Concentrations of Σ_19_PCBs in (a) grape peels, (b) grape pulps and (c) leaves in the control area (CA) and experimental area (EA) before and after BM application. The details of each sample were described in Table A in S1 File.

The levels of PCBs in grape peels, pulps and leaves in LA, CA and EA during the whole experimental period were shown in Fig 3. The Σ_19_PCBs was 48.0 pg/g fresh weight (fw) (P10~P12) in grapes from the experimental area, which was comparable with those from the earlier un-sprayed grape peels (average 54.2 pg/g fw, P1~P6) and those from control area (average 52.4 pg/g fw, P7~P9). The PCB levels of pulps were much lower than that in corresponding peels, showing an average of 10.1 pg/g fw (U1~U12). The same trend was observed by Müller et al., who proved compounds in the peels could be 4~8 times higher than pulps [22]. The total PCB levels in grape pulps and peels collected before and after BM application showed no notable changes (Fig 3). However, the BM application obviously influenced the total PCB levels in the grape leaves. The Σ_19_PCBs in sprayed leaves (EA: average 250 pg/g fw) were obviously higher than those in the un-sprayed leaves (CA: average 174 pg/g fw). On the other hand, it was also very clear that PCB levels increased from 70.8 pg/g fw in the first time sampling leaves to 174 pg/g fw in the second time samples, with a time-span of 53 days. Several studies have reported leaf plants to be the “sink” of PCBs. Aslan et al. conducted a series of investigations among plant products, finding that contaminants in most of the plants were below the detection limits, except for the leafy vegetables [23]. The plant cuticles, especially those with leaf waxes, enabled the leaf plant to absorb airborne lipophilic compounds [24]. A large specific area of leaves would also be helpful. Not surprisingly, both tree barks [25–26] and hulls [27] have been reported as persistent organic pollutant indicators due to their relatively coarse surface. Obviously, the surface of grape leaves were much coarser than that of grape peels; thus, the leaves were a better indicator of PCBs than grape peels.

Environmental factors (air and soil) were also evaluated to determine their potential influence on PCB levels in grapes. The concentrations of PCBs in air samples were in the range of 17.1~27.5 pg/m^3^ (Table D in S1 File). This was consistent with a previous study in Beijing [28] and a little lower than the air samples collected from the urban area but comparable to those from the rural area [29]. Air samples collected from July 28th~November 5th showed a little decrease compared to those collected from April 19th~July 28th. PCBs were prone to exist in the air-phase when the temperature was high [29]. According to the temperature records in 2013, it was a normal distribution curve, and the peak appeared on August 17th. As a result, the average higher concentration was observed in the first sampling period.

Among the 9 soil samples, the PCBs were in the range of 54.8~90.6 pg/g in seven samples, but there were still two soils that weighed 282 and 343 pg/g. It implied that some soil with high PCB concentrations existed in the vineyard. With respect to the background levels of PCBs in the soils of this vineyard, these soils were most likely to be polluted by BM at some particular point. Lower PCB concentrations were also observed in soil samples collected in July 28th, which was consistent with PCB levels in the air. The PCBs evaporated into the air rather than existing in the soil during this period due to the temperature-dependent soil-air balance of PCBs.

### 3.3. Source Analysis

In present study, the PCBs might be introduced to grapes through three potential pathways: atmospheric deposition, root absorption, and BM application. Because very large discrepancies of PCB levels were observed between different sample matrices, the congener ratios were employed to calculate the correlations. Pearson’s correlation coefficient was conducted to evaluate the bivariate relationships between grape products and environmental factors (Table 1). PCBs in the air and soils were strongly correlated with grape products. Grape peels were strongly correlated with the air (R = 0.876, *p*<0.001) and soil (R = 0.841, *p*<0.001). A significant correlation coefficient (R = 0.991, *p*<0.001) also has been observed between the PCBs in grape pulps and in soils; however, it was surprising that neither PCBs in grape peels or in grape pulps were correlated with PCBs in BM (R = 0.262, *p* = 0.279). It had been suggested that the main uptake pathway of PCBs in fruits was the adsorption and adhesion of PCBs in the air phase rather than via the roots [30], and only crops of the cucumber family can absorb dioxin-like compounds from the root [31]. This theory is different from our findings. The PCB content in the leave samples showed an obvious increase after BM application. Both air deposition and BM application were responsible for the PCB increase. PCB congener ratios between the leaves and air were strongly correlated (R = 0.976, *p*<0.001). Though the correlation of PCBs between the leaves and BM samples were not significant (R = 0.312, *p* = 0.193), certain congeners exhibited an obvious increase after BM application (e.g., CB77 increased from 5.35 to 8.48 pg/g fw, CB126 increased from 0.84 to 5.56 pg/g fw). These congeners were recognized as the main components of PCBs in CuSO_4_, suggesting an influence of BM application on the PCB levels in leaves. What’ more, an increase of the most toxic congener CB 126 was also observed between the control and experimental groups in grape peels. This implied that the main contributor (CB 126: from 1.66 to 2.93 pg/g fw) in CuSO_4_ also had an influenced on edible part of grapes.

**Table 1:** Pearson’s correlation matrix of grape products and environmental factors^a^.

	air	soil	grape peels	grape pulps	leaves
soil	0.989*				
grape peels	0.876*	0.841*			
grape pulps	0.998*	0.991*	0.874*		
leaves	0.976*	0.977*	0.794*	0.979*	
BM	0.245	0.255	0.137	0.262	0.312

^a^ The symbol * represent a significant correlation at the *p* < 0.001 level.

Air and soil were the major storage and transport media for PCBs in the environment, respectively. Many previous publications have argued that direct atmospheric deposition would be the predominant source of PCDD/Fs and PCBs in fruit and vegetation [30, 32–34]. Soil is considered to be the second source and plays a subordinate role by being the sink of semi-volatile compounds that allows them to re-suspend into air, then reach balance with air, and subsequently, deposit on plants [24]. PCBs in air and soil samples were strongly correlated (R = 0.989, *p*<0.001).

**Fig 4:**
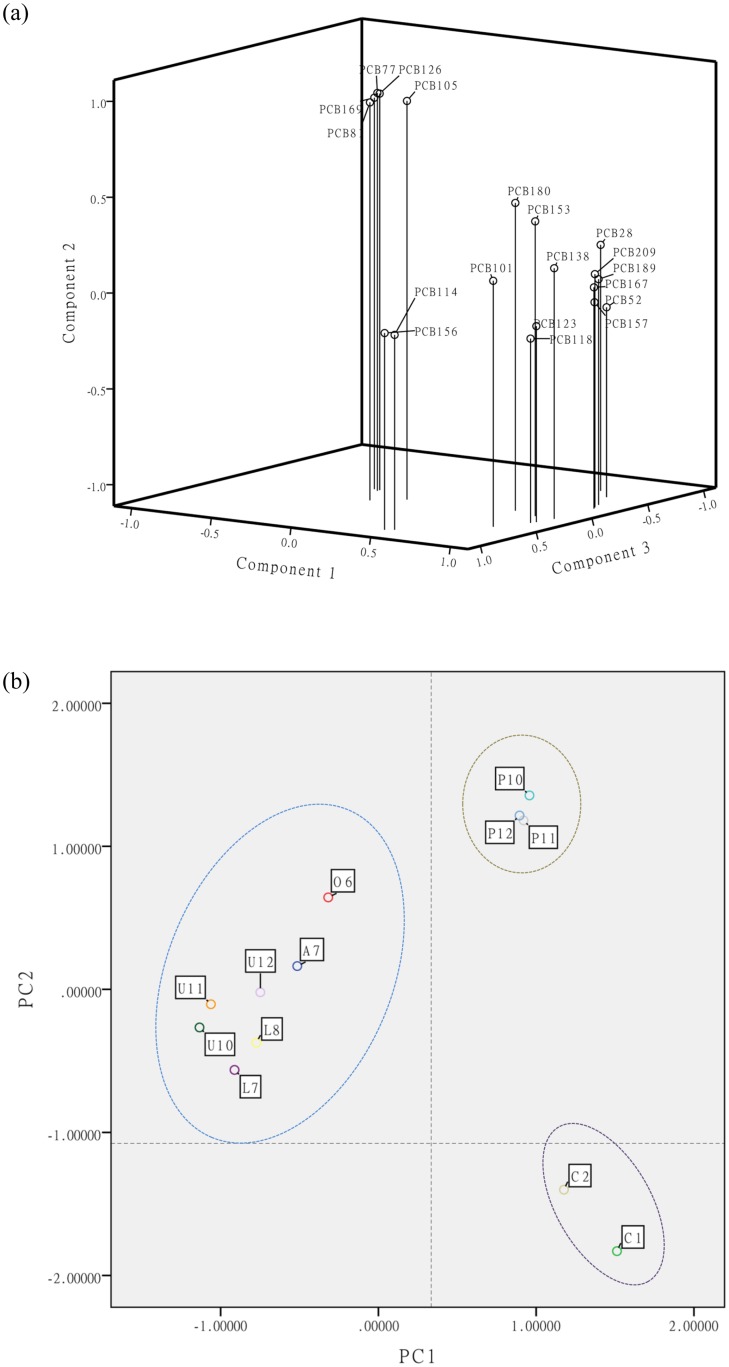
The (a) loading plot and (b) score plot of the principle component analysis (PCA) of PCBs in field grape peels, grape pulps, grape leaves, air, soil and copper sulfate samples after BM application. C1 and C2 were the CuSO_4_ used in the field experiment; A7 was the passive air sampler in experimental area during July 28th~November 5^th^; P10-P12, U10-U12 and L7-L8 were the grape peels, pulps and leaves obtained after BM spray in experimental area respectively.

A principal component analysis was employed to examine the factors that might affect the PCB levels in grape products. Fig 4 presents the loading and score plot using the data of copper sulfates (C1~C2), grape products after BM spray (peels: P10~P12; pulps: U10~U12; leaves: L7~L8) and the corresponding environmental factors (air: A7; soil: O6). The first three components represented a total of 95.3% of the cumulative variances in the Σ_19_PCB concentration. Component 1 and component 2 accounted for 51.5% and 17.3% of the total variance, respectively, which indicates that the selected principle components were suitable and efficient to describe the characteristics of these samples. The grouping of PCBs were mainly determined by their contribution in different matrices. High levels of PCBs in CuSO_4_ were found clustering in the top left corner, and the main contributors of PCBs in the grape products and environmental factors were found in the positive direction of component 1, middle position of component 2 and negative direction of component 3. In the score plot, the data were clearly grouped by three clusters, which suggested that PCBs in BM, grape pulps and other matrices may come from different sources. Both Fig 4(b) and Table 1 indicated that direct exposure to environmental factors was a major influence on grape products.

The soils of vineyards were considered to be seriously contaminated by heavy metals because of the wide application of copper-based pesticides [35–37]; however, because there have been no studies investigating the PCB contamination in vineyards yet, then the PCB contamination in vineyard soil might have been overlooked to a great extent. Take our field experiment for example: BM was sprayed three times a year, and approximately 500 kg of BM was sprayed each time per hectare. According to the PCB contents of C1 and C2 (average 860 pg/g), the absolute amount of PCBs discharged in this vineyard reached 12,905 ng per hectare every year. It should be noted that the grapevines were covered with soil to keep in the temperature and humidity, which protected them to throughout the winter. During this process, the soils must be plowed deeply, and the surface soils were mixed with deep soils, which were in favor of chemical diffusion in vertical and parallel aspects. Sometimes, even the surface soil showed a relatively low concentration of PCBs, and the soil over one meter deep might have already been polluted. The grapevine-bury action took place annually, which would lead to the accumulation of contaminants in the local area and possess increasing potential health risk. This is a problem that should arouse more public concern.

### 3.4. PCBs in National Market Grapes

The concentrations and WHO-TEQ_2005_ of PCBs in national grapes are listed in Table 2 and Table B in S1 File. The concentrations of Σ_19_PCBs and the TEQ in the whole grape were in the range of 20.5~1165 pg/g, and 0.004~0.22 pg WHO-TEQ/g, with mean values of 168 pg/g and 0.05 pg WHO-TEQ/g, respectively. The average concentration of PCBs were relatively higher than that in our field experiment (average Σ_19_PCBs: 52.2 pg WHO-TEQ/g), but the TEQ values were comparable (TEQ: 0.04 pg WHO-TEQ/g). Certain of these national grape samples contained even higher levels of PCBs than those from the industrial area (Σ_26_PCBs: 767 pg/g fw, TEQ: 0.01 pg TEQ/g) [24] and the municipal waste incinerators area (TEQ: 0.0043 pg TEQ/g) [23].

**Table 2:** Concentrations and WHO-TEQs of PCBs in national market grape samples (n = 27).

Congeners	Mass concentration (pg/g)						WHO-TEQ_2005_ (fg TEQ/g)				
	Range	Mean±SD	Median	90^th^ percentile	Percent contribution (%)	Detection ration (%)	Range	Mean±SD	Median	90^th^ percentile	Percent contribution (%)
PCB-77	0.22–7.31	2.32±1.99	1.39	5.93	1.37	100	0.02–0.73	0.22±0.19	0.14	0.49	0.49
PCB-81	nd*-1.90	0.34±0.37	0.28	0.56	0.20	96.3	nd-0.57	0.10±0.11	0.07	0.17	0.22
PCB-105	0.34–28.9	5.08±6.49	2.30	13.5	3.00	100	0.01–0.44	0.10±0.11	0.07	0.21	0.23
PCB-114	0.07–2.36	0.59±0.58	0.36	1.37	0.35	100	0.002–0.07	0.02±0.01	0.01	0.03	0.03
PCB-118	0.68–72.6	12.3±16.2	4.73	32.8	7.22	100	0.02–1.01	0.24±0.26	0.13	0.51	0.54
PCB-123	0.09–8.21	1.45±1.81	0.75	3.87	0.85	100	0.003–0.12	0.03±0.03	0.02	0.05	0.07
PCB-126	0.03–2.09	0.41±0.43	0.28	0.71	0.24	100	3.40–209.1	41.1±42.8	27.9	74.6	92.8
PCB-156	0.07–5.39	1.09±1.24	0.62	2.63	0.64	100	0.002–0.10	0.03±0.02	0.02	0.05	0.06
PCB-157	0.02–1.34	0.29±0.32	0.19	0.64	0.17	100	0.001–0.03	0.01±0.01	0.01	0.01	0.02
PCB-167	0.03–2.54	0.45±0.54	0.24	1.14	0.27	100	0.001–0.04	0.01±0.01	0.01	0.02	0.02
PCB-169	nd-0.39	0.09±0.10	0.07	0.22	0.05	88.9	nd-11.7	2.45±2.65	2.04	4.74	5.48
PCB-189	nd-0.70	0.17±0.17	0.12	0.35	0.10	88.9	nd-0.02	0.01±0.01	0.004	0.01	0.01
PCB-28	9.52–832	87.3±172	28.0	148	51.5	100					
PCB-52	1.92–149	18.0±31.5	6.36	44.9	10.6	100					
PCB-101	nd-68.3	4.83±15.2	0.00	12.1	2.85	11.1					
PCB-138	0.97–67.1	16.2±17.0	10.7	37.1	9.53	100					
PCB-153	0.94–54.7	14.8±15.2	8.89	35.8	8.77	100					
PCB-180	nd-10.7	2.06±3.05	0.51	7.01	1.21	85.2					
PCB-209	0.24–8.16	1.88±1.81	1.19	4.12	1.11	100					
∑_12_ PCBs	1.62–129	24.5±29.3	13.0	63.5			3.67–224	44.6±45.9	31.4	80.9	
∑_19_ PCBs	20.5–1165	170±245	84.6	385							

nd: not detected.

As might be expected, the highest PCBs were found in grapes collected from Taizhou in Zhejiang province (S21~S24). This city was notorious for PCB contamination due to the extensive e-waste dismantling activities in the past decades. Though levels of PCBs have decreased in the past few years due to the strict regulations of open combustion activities by local government, the PCB levels were still relatively high in this area [4,27]. The Σ_19_PCBs and TEQ contents in grape samples from this area ranged from 1165 to 3319 pg/g and from 0.22 to 0.59 pg TEQ/g, respectively. The average PCBs contents and TEQ values in these four grape samples were much higher than that in the rest of national market samples (1,897 pg/g vs 101 pg/g and 0.40 pg TEQ/g vs 0.03 pg TEQ/g, respectively).

The PCBs in the grapes from the field experiment and market might have the same sources. Principal component analysis (Fig B in S1 File) showed a cluster of field experimental grapes (P1~P12) and market grapes (S1~S27). Component 1 contributed greatly (90.5%) to all the samples, and it almost represented the principal component. We made a comparison between the environmental factors obtained from our field experiments and a national investigation of PCBs in air and soil [38]. The congener profiles of PCBs in air and soil were described in Fig C in S1 File. It noted that the homologue distributions of PCBs in the air from the field experiment were quite similar to the National Air Monitoring Project. Tri-CBs were the main congeners. It was consistent with the fact that 90% PCBs produced during 1965~1974 were tri-chlorobiphenyls [8–9]. Grapes collected from the PCB-contaminated area (e-waste dismantling area) also supported the conclusion that higher levels of PCBs in the environment were responsible for higher PCB levels in grapes.

### 3.5. Risk Assessment

Risk assessment was carried out to evaluate whether the observed PCB levels in the grapes posed a potential risk to the consumers. As it was known that people often washed grapes before they ate, we simulated the washing process in several grape samples. The results showed that Σ_19_PCB levels slightly decreased from 102 to 86.3 pg/g. It seemed that a fresh water wash was not very helpful in removing PCBs from grapes.

The estimated daily intake (EDI) was calculated by [EDI (pg kg^-1^ day^-1^) = grape consumption (g kg^-1^ day^-1^)×PCB concentration (pg/g)], with an assumption that the average body weight of Chinese people was 60 kg, and the daily consumption of grapes was 250 g. Therefore, the EDI ranged from 0.09~13.8 ng kg^-1^ day^-1^ and 0.02~2.48 pg TEQ/kg body weight, with average values of 1.62 ng kg^-1^ day^-1^ and 0.38 pg TEQ/kg body weight, respectively. According to the limitation of the WHO (1~4 pg TEQ/kg body weight ingestion) [39], the results of the present research indicated that the intake of PCBs from grapes were not harmful to humans; However, the PCB ingestion through grapes from Taizhou represented 46.5% of the acceptable daily intake of TEQ (without PCDD/Fs contribution). Seike et al. has investigated the enrichment of PCBs in 9 different fruits, among which grapes showed the highest TEQ values [30]. Therefore, a special precaution still should be taken with the possible excessive exposure through grapes from an environmentally contamination area, though the results in this work show a negligible risk associated with exposure via grape consumption.

## Conclusions

To investigate the PCB and PCDD/F contamination status in CuSO_4_ and the potential influence of these chemicals on grapes due to the wide usage of CuSO_4_ as a fungicide in the grape industry, a total of sixty-two field samples (including agricultural-grade and analytical-grade copper sulfates, air, soil, grape peels, grape pulps and grape leaves) and twenty-seven market grape samples were collected and analyzed in this study. High levels of PCBs were first found in copper sulfates, and strong correlations were found among all the agricultural-grade CuSO_4_. A field experiment indicated that these PCB-contaminated CuSO_4_ might influence PCBs on grape leaves, though the environmental factors (air and soil) might be considered as the main contributors for the PCBs in grape products. The PCB levels were highest in grapes collected from the PCB-contaminated area in our national survey, which also suggested that environmental PCBs were the primary contributors to grape contamination. Risk assessment indicated that the intake of PCBs from the grapes were generally under the WHO limitation; however, grapes from polluted areas should be given special attention. Plants with edible leaves that use CuSO_4_ as fungicides should be investigated in the future.

## Supporting Information

S1 File All of the supporting tables and figures were listed in the S1 File. It includes information on the sample IDs, sample descriptions, sampling dates and sample numbers in the field experiment (**Table A**); Concentrations of PCBs in grapes from national markets and e-waste dismantling area (**Table B**); Correlations among PCB homologues in copper sulfates of field experiment (C1~C2) samples and agricultural grade (C3~C10) samples (**Table C**); The concentrations of Σ_19_PCBs in air and soil samples from different functional zones and sampling time (**Table D**); The homologue distribution of PCBs in CuSO4 of field experiment (C1~C2), agricultural grade CuSO4 (C3~C10) and analytical grade CuSO4 (C11~C14) (**Fig A**); Loading plot of Principle component analysis (PCA) of PCBs in national grape samples (**Fig B**); Congener profiles of PCBs in (a) air, (b) soil samples and (c) national air survey (**Fig C**).
(DOCX)
