# Supplementary material for: Assessment of the Polychlorinated Biphenyl (PCB) Occurrence in Copper Sulfates and the Influential Role of PCB Levels on Grapes
Source: PLoS One. 2015 Dec 14;10(12):e0144896. doi: 10.1371/journal.pone.0144896 (PMC4682808; doi:10.1371/journal.pone.0144896)
Supplement: S1 File — It includes information on the sample IDs, sample descriptions, sampling dates and sample numbers in the field experiment (Table A); Concentrations of PCBs in grapes from national markets and e-waste dismantling area (Table B); Correlations among PCB homologues in copper sulfates of field experiment (C1~C2) samples and agricultural grade (C3~C10) samples (Table C); The concentrations of Σ19PCBs in air and soil samples from different functional zones and sampling time (Table D); The homologue distribution of PCBs in CuSO4 of field experiment (C1~C2), agricultural grade CuSO4 (C3~C10) and analytical grade CuSO4 (C11~C14) (Fig A); Loading plot of Principle component analysis (PCA) of PCBs in national grape samples (Fig B); Congener profiles of PCBs in (a) air, (b) soil samples and (c) national air survey (Fig C). (DOCX) [file pone.0144896.s001.docx]

**Supporting Information**

Assessment of the polychlorinated biphenyl (PCB) occurrence in copper sulfates and the influential role of PCB levels on grapes

Xiaomin Li*, Xiaoou Su*

Institute of Quality Standard and Testing Technology for Agro-Products, The Chinese Academy of Agricultural Sciences (CAAS), Beijing 100081, China

* Corresponding author:

Tel.: +86 10 82106576

Fax: +86 10 82106580

E-mail address: lixiaomin@caas.cn

Table A. Information on the sample IDs, sample descriptions, sampling dates and sample numbers in the field experiment.

|  | Sample ID | Description | Sampling Date | Sample numbers (n) |
| --- | --- | --- | --- | --- |
| Air | A1 | travelling blank | April 19th~July 28th | 1 |
|  | A2~A4 | before BM application | April 19th~July 28th | 3 |
|  | A5~A7 | after BM application | July 28th~November 5th | 3 |
| Soil | O1~O3 | living, control and experimental area | April 19th | 3 |
|  | O4~O6 | living, control and experimental area | July 28th | 3 |
|  | O7~O9 | living, control and experimental area | November 5th | 3 |
| Grape peels | P1~P6 | control and experimental area samples before BM application | July 28th | 6 |
|  | P7~P12 | control and experimental area samples after BM application | September 21st | 6 |
| Grape pulps | U1~U6 | control and experimental area samples before BM application | July 28th | 6 |
|  | U7~U12 | control and experimental area samples after BM application | September 21st | 6 |
| Leaves | L1~L4 | control and experimental area samples before BM application | July 28th | 4 |
|  | L5~L8 | control and experimental area samples after BM application | September 21st | 4 |
| Copper sulfates | C1~C2 | agricultural grade collected from vineyard in field experiment | - | 2 |
|  | C3~C10 | agricultural grade collected from markets | - | 8 |
|  | C11~C14 | analytical grade | - | 4 |
| TOTAL |  | | | 62 |

Table B Concentrations of PCBs in grapes from national markets and e-waste dismantling area.

|  | Discription | Production place | Σ_19_PCBs  pg/g | Σ_12_PCBs  pg/g | TEQ  pg WHO-TEQ/g |
| --- | --- | --- | --- | --- | --- |
| S1 | primary production regions | Xiangshan, Beijing | 125.15 | 17.20 | 0.032 |
| S2 |  | Changping, Beijing | 47.04 | 8.79 | 0.035 |
| S3 |  | Yanqing, Beijing | 46.73 | 9.37 | 0.031 |
| S4 |  | Kunming, Yunnan | 156.93 | 35.09 | 0.076 |
| S5 |  | Xinxiang, Henan | 101.03 | 27.63 | 0.074 |
| S6 |  | Kaifeng, Henan | 117.25 | 26.69 | 0.048 |
| S7 |  | Handan, Hebei | 251.28 | 65.29 | 0.079 |
| S8 |  | Xuanhua,Hebei | 67.47 | 15.36 | 0.074 |
| S9 |  | Zhangjiakou, Hebei | 65.33 | 8.98 | 0.023 |
| S10 |  | Weifang, Shandong | 160.28 | 6.02 | 0.009 |
| S11 |  | Yantai, Shandong | 36.41 | 3.64 | 0.007 |
| S12 |  | Rizhao, Shandong | 36.99 | 3.86 | 0.011 |
| S13 |  | Xianyang, Shanxi | 43.81 | 3.66 | 0.011 |
| S14 |  | Xian, Shanxi | 91.27 | 12.97 | 0.066 |
| S15 |  | Wulumuqi, Xinjiang | 20.47 | 2.40 | 0.011 |
| S16 |  | Xinjiang | 24.77 | 1.62 | 0.006 |
| S17 |  | Nanjing, Jiangsu | 84.56 | 31.69 | 0.012 |
| S18 |  | Zhenjiang, Jiangsu | 41.89 | 6.92 | 0.010 |
| S19 |  | Wuhan, Hubei | 34.09 | 4.83 | 0.022 |
| S20 |  | Jinhua, Zhejiang | 558.82 | 19.36 | 0.026 |
| S21 | e-waste dismantling area | Taizhou, Zhejiang | 1164.48 | 67.18 | 0.224 |
| S22 |  | Taizhou, Zhejiang | 1523.37 | 348.00 | 0.40 |
| S23 |  | Taizhou, Zhejiang | 3319.26 | 761.21 | 0.59 |
| S24 |  | Taizhou, Zhejiang | 1579.96 | 366.27 | 0.37 |
| S25 | imported | Chile | 40.92 | 8.08 | 0.004 |
| S26 | fresh water wash | S3-wash | 29.99 | 5.68 | 0.018 |
| S27 |  | S4-wash | 142.65 | 19.41 | 0.066 |

Table C Correlations among PCB homologues in copper sulfates of field experiment (C1~C2) samples and agricultural grade (C3~C10) samples.

|  | C1 | C2 | C3 | C4 | C5 | C6 | C7 | C8 | C9 | C10 |
| --- | --- | --- | --- | --- | --- | --- | --- | --- | --- | --- |
| C1 | 1 |  |  |  |  |  |  |  |  |  |
| C2 | .971^**^ | 1 |  |  |  |  |  |  |  |  |
| C3 | .975^**^ | .979^**^ | 1 |  |  |  |  |  |  |  |
| C4 | .781^**^ | .777^**^ | .852^**^ | 1 |  |  |  |  |  |  |
| C5 | .857^**^ | .822^**^ | .888^**^ | .979^**^ | 1 |  |  |  |  |  |
| C6 | .903^**^ | .801^**^ | .843^**^ | .785^**^ | .894^**^ | 1 |  |  |  |  |
| C7 | .956^**^ | .919^**^ | .910^**^ | .613^**^ | .709^**^ | .835^**^ | 1 |  |  |  |
| C8 | .852^**^ | .813^**^ | .880^**^ | .970^**^ | .987^**^ | .883^**^ | .730^**^ | 1 |  |  |
| C9 | .902^**^ | .815^**^ | .866^**^ | .863^**^ | .944^**^ | .985^**^ | .818^**^ | .946^**^ | 1 |  |
| C10 | .858^**^ | .820^**^ | .892^**^ | .967^**^ | .992^**^ | .901^**^ | .736^**^ | .987^**^ | .950^**^ | 1 |
| **. 在 .01 水平（双侧）上显著相关。 | | | | | | | | | | |


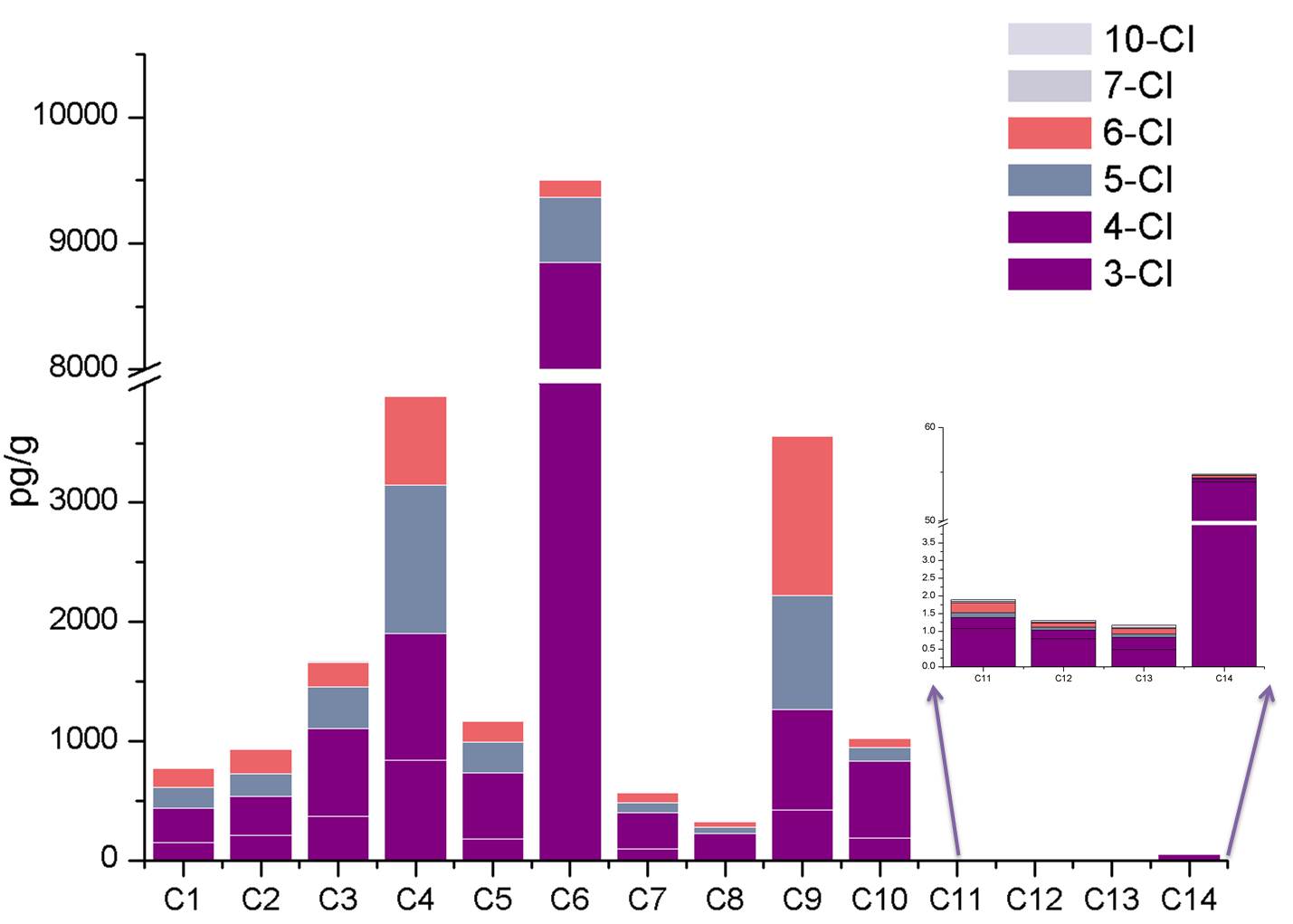


Figure A The homologue distribution of PCBs in CuSO_4_ of field experiment (C1~C2), agricultural grade CuSO_4_ (C3~C10) and analytical grade CuSO_4_ (C11~C14).

Table D The concentrations of Σ_19_PCBs in air and soil samples from different functional zones and sampling time.

|  | CB77 | CB81 | CB105 | CB114 | CB118 | CB123 | CB126 | CB156 | CB157 | CB167 | CB169 | CB189 | CB28 | CB52 | CB101 | CB138 | CB153 | CB180 | CB209 | Σ_19_PCB |
| --- | --- | --- | --- | --- | --- | --- | --- | --- | --- | --- | --- | --- | --- | --- | --- | --- | --- | --- | --- | --- |
|  | Air (pg/m^3^) | | | | | | | | | | | | | | | | | | | |
| A2 | 0.36 | 0.10 | 0.72 | 0.11 | 1.71 | 0.22 | 0.07 | 0.19 | 0.05 | 0.08 | 0.01 | 0.04 | 14.2 | 3.45 | 2.27 | 1.73 | 1.57 | 0.37 | 0.25 | 27.5 |
| A3 | 0.30 | 0.09 | 0.63 | 0.10 | 1.51 | 0.23 | 0.06 | 0.15 | 0.05 | 0.05 | 0.01 | 0.04 | 12.9 | 3.10 | 2.06 | 1.57 | 1.44 | 0.35 | 0.20 | 24.8 |
| A4 | 0.28 | 0.06 | 0.60 | 0.10 | 1.40 | 0.19 | 0.06 | 0.15 | 0.05 | 0.05 | 0.01 | 0.04 | 11.8 | 2.88 | 1.85 | 1.44 | 1.33 | 0.32 | 0.29 | 22.9 |
| A5 | 0.18 | 0.03 | 0.54 | 0.06 | 1.35 | 0.18 | 0.03 | 0.11 | 0.03 | 0.05 | 0.00 | 0.02 | 9.46 | 2.54 | 1.72 | 1.34 | 1.75 | 0.26 | 0.16 | 19.8 |
| A6 | 0.18 | 0.04 | 0.53 | 0.06 | 1.30 | 0.14 | 0.02 | 0.12 | 0.03 | 0.04 | 0.01 | 0.01 | 9.31 | 3.19 | 1.86 | 1.20 | 1.13 | 0.24 | 0.11 | 19.5 |
| A7 | 0.15 | 0.05 | 0.47 | 0.04 | 1.13 | 0.11 | 0.03 | 0.10 | 0.02 | 0.05 | 0.01 | 0.02 | 8.74 | 2.32 | 1.44 | 1.04 | 1.04 | 0.23 | 0.14 | 17.1 |
|  | Soil (pg/g) | | | | | | | | | | | | | | | | | | | |
| O1 | 0.81 | 0.13 | 0.86 | 0.14 | 1.46 | 0.19 | 0.16 | 0.35 | 0.09 | 0.08 | 0.10 | 0.16 | 51.7 | 13.2 | 3.11 | 2.83 | 2.59 | 0.89 | 5.80 | 84.6 |
| O2 | 1.19 | 0.23 | 1.51 | 0.27 | 3.26 | 0.57 | 0.21 | 0.50 | 0.17 | 0.15 | 0.09 | 0.14 | 164 | 74.0 | 14.52 | 7.75 | 7.83 | 1.97 | 3.91 | 282 |
| O3 | 0.93 | 0.13 | 1.06 | 0.12 | 2.09 | 0.46 | 0.19 | 0.43 | 0.12 | 0.09 | 0.08 | 0.28 | 39.1 | 9.96 | 4.32 | 6.27 | 5.94 | 1.71 | 3.60 | 76.9 |
| O4 | 3.23 | 0.10 | 0.96 | 0.14 | 1.69 | 0.26 | 0.18 | 0.31 | 0.11 | 0.06 | 0.06 | 0.19 | 27.4 | 6.23 | 4.04 | 3.23 | 2.82 | 0.90 | 5.01 | 57.0 |
| O5 | 0.80 | 0.11 | 0.95 | 0.10 | 1.86 | 0.30 | 0.20 | 0.41 | 0.13 | 0.13 | 0.08 | 0.24 | 27.7 | 7.54 | 4.03 | 6.33 | 5.79 | 1.68 | 3.61 | 62.0 |
| O6 | 0.73 | 0.14 | 0.87 | 0.16 | 1.99 | 0.40 | 0.20 | 0.41 | 0.14 | 0.13 | 0.10 | 0.29 | 26.4 | 6.41 | 4.00 | 6.42 | 5.64 | 1.68 | 3.51 | 59.5 |
| O7 | 0.62 | 0.12 | 0.71 | 0.13 | 1.44 | 0.29 | 0.16 | 0.26 | 0.11 | 0.06 | 0.08 | 0.14 | 29.4 | 7.99 | 2.84 | 2.85 | 2.63 | 0.87 | 4.08 | 54.8 |
| O8 | 1.07 | 0.22 | 1.68 | 0.25 | 3.68 | 0.58 | 0.22 | 0.31 | 0.11 | 0.12 | 0.07 | 0.24 | 205 | 91.7 | 16.11 | 7.25 | 7.95 | 1.54 | 4.58 | 343 |
| O9 | 0.81 | 0.15 | 1.05 | 0.20 | 2.14 | 0.52 | 0.22 | 0.46 | 0.18 | 0.11 | 0.07 | 0.36 | 44.4 | 15.7 | 5.68 | 6.69 | 6.38 | 1.74 | 3.64 | 90.6 |

^*^  LA: the living area; CA: the control area; EA: the experimental area.


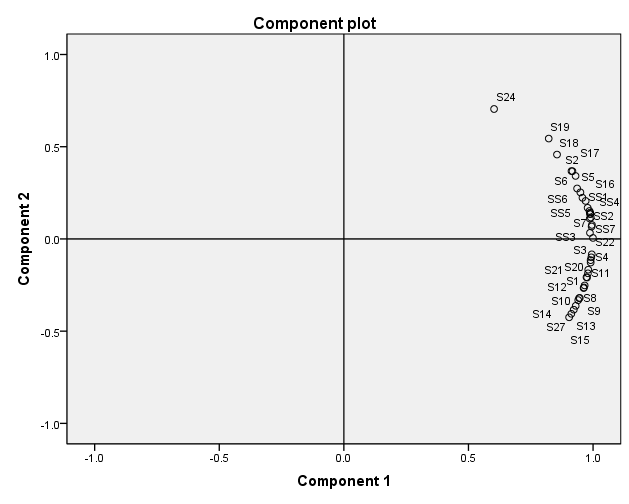


Figure B Loading plot of Principle component analysis (PCA) of PCBs in national grape samples.


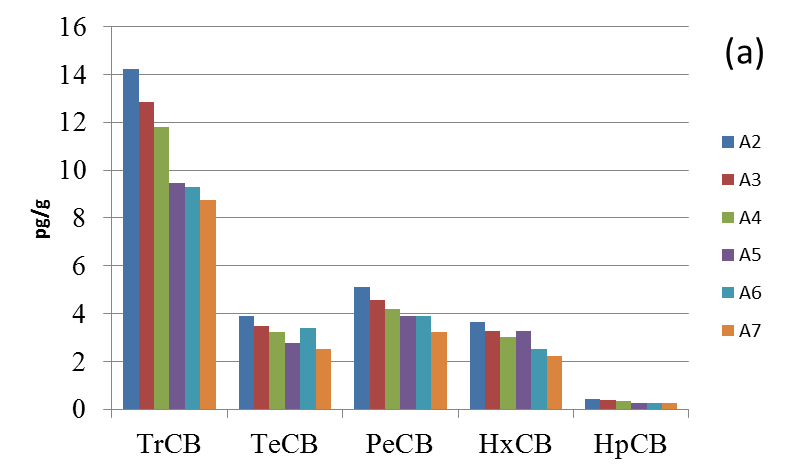


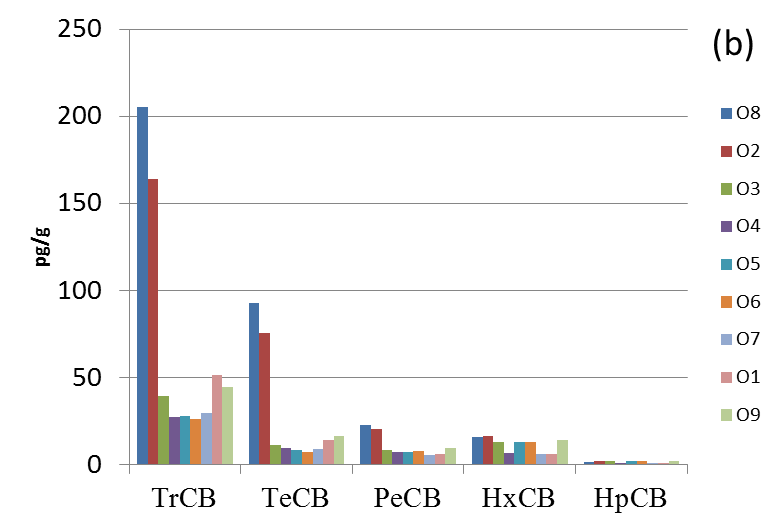


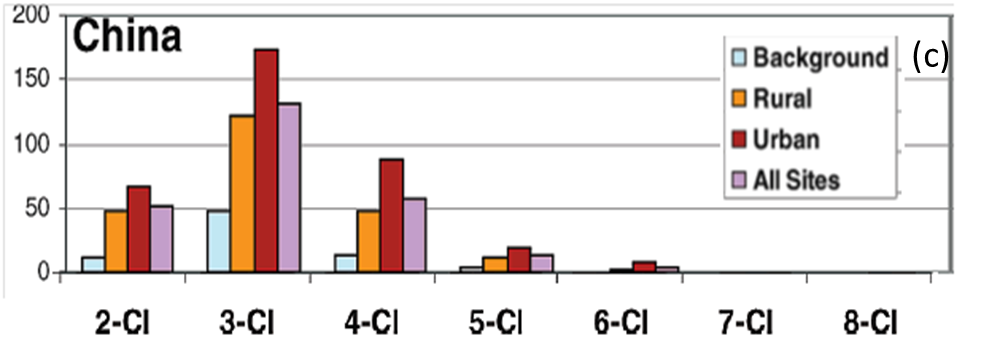


Figure C Congener profiles of PCBs in (a) air, (b) soil samples and (c) national air survey. Fig C(c) was available from a previous work. (Zhang et al., 2008)

Zhang, Z., Liu, L., Li, Y.-F., Wang, D., Jia, H., Harner, T., Sverko, E., Wan, X., Xu, D., Ren, N., Ma, J., Pozo, K., 2008. Analysis of polychlorinated biphenyls in concurrently sampled Chinese air and surface soil. Environ. Sci. Technol. 42, 6514-6518.
